# Anthropometric and reproductive factors and risk of esophageal and gastric cancer by subtype and subsite: Results from the European Prospective Investigation into Cancer and Nutrition (EPIC) cohort

**DOI:** 10.1002/ijc.32386

**Published:** 2019-05-21

**Authors:** Harinakshi Sanikini, David C. Muller, Marisa Sophiea, Sabina Rinaldi, Antonio Agudo, Eric J. Duell, Elisabete Weiderpass, Kim Overvad, Anne Tjønneland, Jytte Halkjær, Marie‐Christine Boutron‐Ruault, Franck Carbonnel, Iris Cervenka, Heiner Boeing, Rudolf Kaaks, Tilman Kühn, Antonia Trichopoulou, Georgia Martimianaki, Anna Karakatsani, Valeria Pala, Domenico Palli, Amalia Mattiello, Rosario Tumino, Carlotta Sacerdote, Guri Skeie, Charlotta Rylander, María‐Dolores Chirlaque López, Maria‐Jose Sánchez, Eva Ardanaz, Sara Regnér, Tanja Stocks, Bas Bueno‐de‐Mesquita, Roel C.H. Vermeulen, Dagfinn Aune, Tammy Y.N. Tong, Nathalie Kliemann, Neil Murphy, Marc Chadeau‐Hyam, Marc J. Gunter, Amanda J. Cross

**Affiliations:** ^1^ Department of Epidemiology and Biostatistics, School of Public Health Imperial College London London United Kingdom; ^2^ Section of Nutrition and Metabolism International Agency for Research on Cancer Lyon France; ^3^ Unit of Nutrition and Cancer, Cancer Epidemiology Research Program Catalan Institute of Oncology (ICO‐IDIBELL) Barcelona Spain; ^4^ Department of Community Medicine, Faculty of Health Sciences University of Tromsø, The Arctic University of Norway Tromsø Norway; ^5^ Department of Research, Cancer Registry of Norway Institute of Population‐Based Cancer Research Oslo Norway; ^6^ Department of Medical Epidemiology and Biostatistics Karolinska Institutet Stockholm Sweden; ^7^ Genetic Epidemiology Group, Folkhälsan Research Center, Faculty of Medicine University of Helsinki Helsinki Finland; ^8^ Department of Public Health Aarhus University Aarhus Denmark; ^9^ Danish Cancer Society Research Center Copenhagen Denmark; ^10^ CESP, Faculté de Médecine Université Paris‐Sud Villejuif France; ^11^ Faculté de Médecine UVSQ, INSERM, Université Paris‐Saclay Villejuif France; ^12^ Institut Gustave Roussy Villejuif France; ^13^ Department of Gastroenterology Bicêtre University Hospital, Assistance Publique des Hôpitaux de Paris Le Kremlin‐Bicêtre France; ^14^ Department of Epidemiology German Institute of Human Nutrition, Potsdam‐Rehbrücke Nuthetal Germany; ^15^ Division of Cancer Epidemiology German Cancer Research Center (DKFZ) Heidelberg Germany; ^16^ Hellenic Health Foundation Athens Greece; ^17^ Pulmonary Medicine Department School of Medicine, National and Kapodistrian University of Athens, “ATTIKON” University Hospital Haidari Greece; ^18^ Epidemiology and Prevention Unit, Department of Research Fondazione IRCCS Istituto Nazionale dei Tumori Milan Italy; ^19^ Cancer Risk Factors and Life‐Style Epidemiology Unit Institute for Cancer Research, Prevention and Clinical Network‐ISPRO Florence Italy; ^20^ Department of Clinical Medicine and Surgery Federico II University Naples Italy; ^21^ Cancer Registry and Histopathology Department "Civic ‐ M. P. Arezzo" Hospital, ASP Ragusa Italy; ^22^ Unit of Cancer Epidemiology Città della Salute e della Scienza University‐Hospital and Center for Cancer Prevention (CPO) Turin Italy; ^23^ Department of Epidemiology Regional Health Council, IMIB‐Arrixaca Murcia Spain; ^24^ CIBER in Epidemiology and Public Health (CIBERESP) Madrid Spain; ^25^ Department of Health and Social Sciences Murcia University Murcia Spain; ^26^ Escuela Andaluza de Salud Pública, Instituto de Investigación Biosanitaria ibs, GRANADA, Hospitales Universitarios de Granada/Universidad de Granada Granada Spain; ^27^ CIBER de Epidemiología y Salud Pública (CIBERESP) Madrid Spain; ^28^ Navarra Public Health Institute Pamplona Spain; ^29^ IdiSNA Navarra Institute for Health Research Pamplona Spain; ^30^ CIBER Epidemiology and Public Health CIBERESP Madrid Spain; ^31^ Institution of Clinical Sciences Malmö, Skåne University Hospital Lund University Sweden; ^32^ Department of Clinical Sciences Lund University Lund Sweden; ^33^ Department for Determinants of Chronic Diseases (DCD) National Institute for Public Health and the Environment (RIVM) Bilthoven The Netherlands; ^34^ Department of Gastroenterology and Hepatology University Medical Centre Utrecht The Netherlands; ^35^ Department of Social & Preventive Medicine, Faculty of Medicine University of Malaya Kuala Lumpur Malaysia; ^36^ Institute for Risk Assessment Sciences, Utrecht University Utrecht The Netherlands; ^37^ Julius Centre for Public Health Sciences and Primary Care Utrecht University Medical Centre Utrecht The Netherlands; ^38^ Department of Nutrition Bjørknes University College Oslo Norway; ^39^ Department of Endocrinology, Morbid Obesity and Preventive Medicine Oslo University Hospital Oslo Norway; ^40^ Cancer Epidemiology Unit, Nuffield Department of Population Health University of Oxford Oxford United Kingdom

**Keywords:** obesity, reproductive, hormones, esophageal, gastric, cancer

## Abstract

Obesity has been associated with upper gastrointestinal cancers; however, there are limited prospective data on associations by subtype/subsite. Obesity can impact hormonal factors, which have been hypothesized to play a role in these cancers. We investigated anthropometric and reproductive factors in relation to esophageal and gastric cancer by subtype and subsite for 476,160 participants from the European Prospective Investigation into Cancer and Nutrition cohort. Multivariable hazard ratios (HRs) and 95% confidence intervals (CIs) were estimated using Cox models. During a mean follow‐up of 14 years, 220 esophageal adenocarcinomas (EA), 195 esophageal squamous cell carcinomas, 243 gastric cardia (GC) and 373 gastric noncardia (GNC) cancers were diagnosed. Body mass index (BMI) was associated with EA in men (BMI ≥30 *vs*. 18.5–25 kg/m^2^: HR = 1.94, 95% CI: 1.25–3.03) and women (HR = 2.66, 95% CI: 1.15–6.19); however, adjustment for waist‐to‐hip ratio (WHR) attenuated these associations. After mutual adjustment for BMI and HC, respectively, WHR and waist circumference (WC) were associated with EA in men (HR = 3.47, 95% CI: 1.99–6.06 for WHR >0.96 *vs*. <0.91; HR = 2.67, 95% CI: 1.52–4.72 for WC >98 *vs*. <90 cm) and women (HR = 4.40, 95% CI: 1.35–14.33 for WHR >0.82 *vs*. <0.76; HR = 5.67, 95% CI: 1.76–18.26 for WC >84 *vs*. <74 cm). WHR was also positively associated with GC in women, and WC was positively associated with GC in men. Inverse associations were observed between parity and EA (HR = 0.38, 95% CI: 0.14–0.99; >2 *vs*. 0) and age at first pregnancy and GNC (HR = 0.54, 95% CI: 0.32–0.91; >26 *vs*. <22 years); whereas bilateral ovariectomy was positively associated with GNC (HR = 1.87, 95% CI: 1.04–3.36). These findings support a role for hormonal pathways in upper gastrointestinal cancers.

## Abstract 2

**What's new?**

Obesity can change the body's hormone balance, and encourage the onset of cancer. Here, the authors investigated the relationship between obesity, hormones, and esophageal and gastric cancers. Using data from the EPIC cohort, they obtained information about anthropometric and reproductive factors for 476,160 participants. Excess fat around the waist, they found, was associated with esophageal adenocarcinoma and gastric cardia cancer, in women and men. In women, bearing children, as well as younger age at first pregnancy, had an inverse association with certain cancers. Ovariectomy was positively associated with gastric non‐cardia cancer, suggesting involvement of hormone pathways in these malignancies.

**Abbreviations**
BMI: body mass index
CI: confidence intervals
EA: esophageal adenocarcinoma
EPIC: European Prospective Investigation into Cancer and Nutrition
ESCC: esophageal squamous cell carcinoma
GC: gastric cardia
GNC: gastric noncardia
HC: hip circumference
HR: hazard ratio
WC: waist circumference
WHR: waist‐to‐hip ratio
WHtR: waist‐to‐height ratio

## Introduction

Esophageal cancer is the seventh most common and gastric cancer the fifth most common cancer worldwide, with an estimated 572,000 and 1,000,000 cases in 2018, respectively.^1^ These cancers are more common in men than in women and are becoming more prevalent in many regions of the world.^1^ Esophageal cancer can be categorized histologically as esophageal adenocarcinoma (EA) and esophageal squamous cell carcinoma (ESCC) and these subtypes have distinct etiologies. Gastric cancers are predominantly adenocarcinomas but their etiology appears to differ depending on their location in the gastric cardia (GC) or gastric noncardia (GNC). Smoking and alcohol are well‐known risk factors for ESCC, whereas gastroesophageal reflux disease (GERD), smoking and obesity are established risk factors for EA.^2^ Smoking and obesity are also thought to be risk factors for GC, while *Helicobacter pylori* infection and smoking are risk factors for GNC.^3^

The role of obesity in upper gastrointestinal cancers has been previously investigated in a number of epidemiological studies.^4^, ^5^, ^6^, ^7^, ^8^, ^9^, ^10^ Two studies conducted within the European Prospective Investigation into Cancer and Nutrition (EPIC) cohort, reported a positive association between body mass index (BMI) and EA.^4^, ^5^ Two other analyses found a positive association between BMI and risk of EA and GC.^11^, ^12^ In addition, the recent report from the World Cancer Research Fund/American Institute for Cancer Research (WCRF/AICR) concluded that there is “convincing” evidence that BMI increases the risk of EA.^13^ Conversely, an inverse association between BMI and ESCC risk has been reported in several epidemiological studies.^2^, ^4^, ^7^, ^14^ The WCRF/AICR report also concluded that there is “probable” evidence that BMI increases the risk of GC.^13^ However, few epidemiological studies have examined the association between abdominal obesity and risk of esophageal and gastric cancer by subtype or subsite and the published findings are inconsistent.^4^, ^5^, ^8^, ^15^

There are several plausible biological mechanisms underlying the association between obesity and upper gastrointestinal cancers. Obesity promotes GERD and its transition to Barrett's esophagus, which increases the risk of EA and GC.^12^, ^16^ Obesity is also associated with a range of metabolic and endocrinologic abnormalities. In particular, obesity can lead to insulin resistance, where circulating levels of insulin and bioavailable insulin‐like growth factor (IGF) are elevated, leading to stimulation of cell proliferation and downregulation of apoptosis.^17^ Obese individuals also have abnormal circulating levels of adipokines (e.g., higher levels of leptin and lower levels of adiponectin), proinflammatory cytokines (e.g., tumor necrosis factor‐α and interleukin‐6) and endogenous sex steroids, which are synthesized in peripheral adipose tissue and may contribute to cancer development.^17^, ^18^

In addition to the link through obesity, sex hormones could also explain the predominance of both esophageal and gastric cancers in men compared to women. It has been suggested that female sex hormones, particularly estrogens, may protect against the development of esophageal and gastric cancer.^19^, ^20^ Some epidemiological studies have investigated the role of hormonal and reproductive factors in the development of esophageal and gastric cancer risk^21^, ^22^, ^23^, ^24^; however, prospective studies examining these relationships by subtype of esophageal cancer and subsite of gastric cancer are limited, with conflicting results.^25^, ^26^, ^27^, ^28^, ^29^ The association between reproductive factors and gastric cancer was investigated in a previous analysis of the EPIC cohort, which included participants with follow‐up through 2004; this analysis showed a positive association between ovariectomy and gastric cancer risk.^28^ In the present study, not only did we have much longer follow‐up of the EPIC cohort data, and therefore more cases, but also we studied the role of reproductive factors in both esophageal and gastric cancer by subtype and subsite.

The aim of the current study was to investigate both anthropometric and reproductive factors in relation to esophageal and gastric cancer by subtype and subsite, respectively, in a large cohort study with long‐term follow‐up.

## Materials and Methods

### Study population

The EPIC study is an ongoing multicenter prospective cohort study aimed at investigating the association between diet, lifestyle, genetic and environmental factors and the development of cancer and other chronic diseases. The methodological details and rationale of the EPIC study have been described previously.^30^, ^31^ In brief, the cohort comprises of 521,448 men and women, aged 25–70 years, recruited between 1992 and 2000 from 23 centers located in 10 European countries including Denmark, France, Germany, Greece, Italy, Norway, Spain, Sweden, the Netherlands and the United Kingdom. Participants were mostly recruited from the general population with some exceptions: French participants were recruited via health insurance databases; some participants of the Italian and Spanish cohorts were recruited through local blood donor registries; participants of the Utrecht (the Netherlands) and Florence (Italy) cohorts were recruited via breast cancer screening programs; the Oxford (United Kingdom) cohort included a large proportion of vegetarians. All participants signed an informed consent form and the study was approved by the ethical review committees of the International Agency for Research on Cancer (IARC) and EPIC centers.

For our study, we excluded participants with prevalent cancer at recruitment (*n* = 29,332), participants who were lost during follow‐up (*n* = 124), participants for whom no dietary or lifestyle information was available (*n* = 6,259) and participants who were in the top or bottom 1% of the ratio of energy intake to estimated energy requirement (*n* = 9,573). After the exclusions, the final sample available for the analysis included 476,160 participants.

### Diet and lifestyle questionnaires

Usual diet was assessed at recruitment using validated country‐specific dietary questionnaires reflecting intake in the past 12 months. A separate questionnaire on lifestyle factors was used to collect information on smoking and alcohol consumption, education, occupation, reproductive history, family history and physical activity.

### Assessment of anthropometric and reproductive data

Anthropometric measurements including height, weight, waist circumference (WC) and hip circumference (HC) were taken at recruitment by trained health professionals in most EPIC centers, except for most of the Oxford cohort, the Norwegian cohort and approximately two‐thirds of the French cohort, in which height and weight were self‐reported. BMI was computed as weight in kilograms divided by height in meters squared, waist‐to‐hip ratio (WHR) was computed as WC (cm) divided by HC (cm) and waist‐to‐height ratio (WHtR) was computed as WC (cm) divided by height (cm).

Information on reproductive history was collected at recruitment. The following reproductive characteristics were assessed: age at menarche, duration of menstrual cycle, ever been pregnant, age at first pregnancy, number of full‐term pregnancies (parity), number of live‐born children, breastfeeding, menopausal status, age at menopause, menopausal hormone use, oral contraceptive (OC) pill use and ovariectomy. More details on questionnaires can be found elsewhere.^30^, ^31^

### Follow‐up and identification of cancer cases

Participants were followed‐up from study entry until cancer diagnosis, death or end of follow‐up, which is currently up to 2015, whichever came first. Population‐based cancer registries, as well as postal follow‐up questionnaires, are used in most of the countries to identify incident cancer cases. In France, Germany, Greece and Naples (Italy) cancer cases are additionally identified through active follow‐up. Data on mortality and movement of participants are obtained through periodic linkage to regional and national mortality registries. First primary incident esophageal and gastric cancers were coded according to the 10th revision of the International Classification of Diseases (ICD‐10). Esophageal cancer included topography ICD‐O codes C15.0–C15.9; EA was categorized as (ICD‐O morphological codes: 8140, 8141, 8190–8231, 8260–8263, 8310, 8430, 8480–8490, 8560, 8570–8572) and ESCC was categorized as (ICD‐O morphological codes: 8050–8076). Gastric adenocarcinomas included topography ICD‐O codes: C16; GC was classified as ICD‐O code C16.0 and GNC included C16.1–16.6.

### Statistical analysis

Cox proportional hazard models were used to calculate hazard ratios (HRs) and 95% confidence intervals (CIs) for the association between anthropometric and reproductive factors and risk of esophageal and gastric cancer by subtype and subsite, respectively. Age was used as the primary time metric, and entry time was defined as age at enrolment and exit time as age at diagnosis, death or end of follow‐up, whichever occurred first. Models were stratified by age at recruitment and study center.

#### *Anthropometric variables*

To account for different body fat distributions of men and women, we used sex‐specific tertiles for anthropometric variables (height, weight, WC, HC, WHR and WHtR). BMI was classified according to World Health Organization (WHO) categories: underweight (BMI < 18.5 kg/m^2^), normal weight (18.5 ≤ BMI < 25 kg/m^2^), overweight (25 ≤ BMI < 30 kg/m^2^) and obese (≥30 kg/m^2^). Mean and standard deviations or frequencies were calculated for baseline characteristics of study participants stratified by BMI categories. All models were adjusted for smoking status (never smoker; former smoker who stopped ≤10, 11–20 or 20+ years ago; current smoker of 1–15, 16–25 or 26+ cigarettes/day; current or occasional pipe/cigar; smoking unknown/missing), and education level (none, primary school, technical/professional, secondary school or university), while models for ESCC were additionally adjusted for alcohol intake (g/day). We also examined models in which BMI and WHR were mutually adjusted, to estimate whether abdominal obesity was associated with upper gastrointestinal cancers independently of the association with general obesity. In addition, separate models were used in which WC and HC were mutually adjusted for each other. Interactions between anthropometric variables with sex and smoking status were explored by including an interaction term along with the main effect term in the adjusted model. The likelihood ratio test was used to compare models with and without interaction terms.

#### *Reproductive variables*

Reproductive variables were classified into categories as follows: age at menarche (<12, 12–14, >14 years), duration of menstrual cycling (<30, 30–35, >35 years), ever been pregnant (yes/no), age at first pregnancy (<22, 22–26, >26 years), parity (0, 1–2, >2 pregnancies), number of live‐born children (1, 2–3, >3), breastfeeding (yes/no), duration of breastfeeding (<4, 4–10, >10 months), menopausal status (pre/perimenopausal, postmenopausal), age at menopause (<48, 48–51, >51 years), menopausal hormone use (yes/no), duration of menopausal hormone use (<2, ≥2 years), OC pill use (yes/no), duration of OC pill use (<5, ≥5 years) and ovariectomy (no, unilateral, bilateral). We computed mean and standard deviations or frequencies for baseline characteristics in women stratified by OC and menopausal hormone use. Models for EA, GC and GNC were adjusted for smoking status, BMI and educational level, while models for ESCC were additionally adjusted for alcohol intake (g/day).

Tests for linear trend across categories of anthropometric and reproductive variables were performed by assigning the median value to each category as a continuous term in the Cox regression models.

To examine possible reverse causation, we performed sensitivity analyses by excluding esophageal and gastric cancer cases diagnosed in the first 2 years of follow up. Additional sensitivity analyses included restricting the analyses to participants in whom height and weight were measured rather than self‐reported. To examine whether the excluded participants differed from those included, we compared the main baseline characteristics in the included participants (*n* = 476,160) to those participants with no dietary or lifestyle information (*n* = 6,259).

All analyses were conducted using SAS 9.4 software (SAS Institute, Cary, NC) and *p* values <0.05 were considered statistically significant.

## Results

During a mean follow‐up of 14 years, 220 EA (171 men and 49 women), 195 ESCC (101 men and 94 women), 243 GC (163 men and 80 women) and 373 GNC (184 men and 189 women) cases were diagnosed among the 476,160 participants (142,241 men and 333,919 women).

### Anthropometric factors

In the overall cohort, 1.1% of participants were underweight, 37.8% were normal weight, 30.9% were overweight and 12.4% were obese at baseline. Baseline characteristics by BMI categories are presented in Supporting Information Table S1. Obese men and women were slightly older, had a higher WC and HC, lower education level, less likely to be smokers, less physically active and had a higher prevalence of diabetes than normal weight subjects. In addition, obese men had a higher intake of fruits and vegetables.

Several anthropometric variables were positively associated with EA in both men and women (Tables 1 and 2), respectively, including BMI (obese *vs*. normal weight, men: HR 1.94, 95% CI: 1.25–3.03; women: HR 2.66, 95% CI: 1.15–6.19), WC (men: HR 2.39, 95% CI: 1.53–3.73 for >98 *vs*. <90 cm; women: HR 2.81, 95% CI: 1.13–6.96 for WC >84 *vs*. <74 cm), WHR (men: HR 3.21, 95% CI: 1.93–5.34 for >0.96 *vs*. <0.91; women: HR 5.39, 95% CI: 1.74–16.72 for >0.82 *vs*. <0.76) and WHtR (men: HR 2.36, 95% CI: 1.40–3.97 for >0.57 *vs*. <0.51; women: HR 3.50, 95% CI: 1.24–9.93 for >0.52 *vs*. <0.45). In addition, weight and HC were positively associated with EA in men but not in women (Tables 1 and 2).

**Table 1:** Adjusted hazard ratios for esophageal and gastric cancer by subtype and subsite in men (*n* = 142,241) according to anthropometric factors in the EPIC study

	Esophageal cancer				Gastric cancer			
	Adenocarcinoma		Squamous cell carcinoma		Cardia		Noncardia	
Men^1^	Cases	Adjusted HR^2^ (95% CI)	Cases	Adjusted HR^3^ (95% CI)	Cases	Adjusted HR^2^ (95% CI)	Cases	Adjusted HR^2^ (95% CI)
Height (cm)								
<171	49	Reference	40	Reference	49	Reference	87	Reference
171–178	62	1.00 (0.68–1.47)	32	0.64 (0.39–1.05)	69	1.10 (0.75–1.63)	74	1.03 (0.74–1.44)
>178	59	1.30 (0.87–1.96)	29	0.74 (0.44–1.27)	45	0.98 (0.63–1.53)	22	0.49 (0.30–0.81)
Missing	1		–	–	–		1	–
*p*_trend_		0.30		0.21		0.81		0.008
Weight (kg)								
<75	42	Reference	47	Reference	51	Reference	64	Reference
75–84	53	1.15 (0.76–1.73)	26	0.49 (0.30–0.79)	59	1.03 (0.70–1.51)	64	0.91 (0.64–1.30)
>84	75	1.78 (1.21–2.63)	28	0.51 (0.31–0.82)	52	0.98 (0.65–1.45)	53	0.84 (0.58–1.23)
Missing	1	–	–	–	1	–	3	
*p*_trend_		0.006		0.003		0.96		0.68
BMI (kg/m^2^)^4^								
Underweight^5^	–	–	1	–	1	–	1	–
Normal weight	50	Reference	53	Reference	51	Reference	49	Reference
Overweight	83	1.15 (0.80–1.65)	31	0.38 (0.24–0.61)	90	1.22 (0.86–1.75)	99	1.13 (0.79–1.62)
Obese	37	1.94 (1.25–3.03)	16	0.52 (0.28–0.95)	20	0.94 (0.55–1.61)	32	1.03 (0.64–1.65)
Missing	1	–		–	1	–	3	–
*p*_trend_		0.02		0.0004		0.56		0.85
Hip circumference (cm)								
<98	35	Reference	48	Reference	54	Reference	49	Reference
98–103	67	1.75 (1.16–2.64)	30	0.57 (0.36–0.91)	53	0.90 (0.61–1.33)	65	1.11 (0.76–1.62)
>103	52	1.59 (1.02–2.47)	17	0.29 (0.16–0.54)	45	0.90 (0.60–1.37)	51	0.82 (0.54–1.26)
Missing	17	–	6	–	11	–	19	–
*p*_trend_		0.03		0.0002		0.84		0.31
Waist circumference (cm)								
<90	30	Reference	29	Reference	32	Reference	39	Reference
90–98	50	1.46 (0.92–2.32)	36	0.89 (0.54–1.47)	66	1.54 (0.99–2.37)	73	1.32 (0.88–1.97)
>98	75	2.39 (1.53–3.73)	31	0.70 (0.41–1.21)	55	1.41 (0.89–2.22)	55	0.86 (0.55–1.34)
Missing	16	–	5	–	10	–	17	–
*p*_trend_		0.0003		0.42		0.15		0.06
Waist‐to‐hip ratio								
<0.91	21	Reference	18	Reference	25	Reference	32	Reference
0.91–0.96	52	1.72 (1.02–2.90)	36	1.17 (0.65–2.08)	63	1.39 (0.87–2.22)	81	1.42 (0.93–2.17)
>0.96	81	3.21 (1.93–5.34)	41	1.26 (0.70–2.29)	64	1.57 (0.97–2.53)	52	0.90 (0.57–1.43)
Missing	17	–	6	–	11	–	19	–
*p*_trend_		<0.0001		0.74		0.19		0.03
Waist‐to‐height ratio								
<0.51	23	Reference	26	Reference	28	Reference	26	Reference
0.51–0.57	76	1.76 (1.09–2.86)	43	0.80 (0.48–1.31)	83	1.31 (0.84–2.03)	82	1.29 (0.82–2.04)
>0.57	56	2.36 (1.40–3.97)	27	0.69 (0.37–1.26)	42	1.13 (0.68–1.88)	59	1.02 (0.61–1.71)
Missing	16	–	5	–	10	–	17	–
*p*_*trend*_		0.006		0.47		0.44		0.31

1 Sex‐specific tertiles were used in the analyses except for BMI.

2 Stratified on age, center and adjusted for smoking and education level.

3 Stratified on age, center and adjusted for smoking, education level and alcohol intake.

4 Underweight (BMI <18.5), normal weight (18.5 ≤ BMI < 25), overweight (25 ≤ BMI < 30) and obese (BMI ≥30).

5 We excluded underweight group from the analysis due to few number of cases.

**Table 2:** Adjusted hazard ratios for esophageal and gastric cancer by subtype and subsite in women (*n* = 333,919) according to anthropometric factors in the EPIC study

	Esophageal cancer				Gastric cancer			
	Adenocarcinoma		Squamous cell carcinoma		Cardia		Noncardia	
Women^1^	Cases	Adjusted HR^2^ (95% CI)	Cases	Adjusted HR^3^ (95% CI)	Cases	Adjusted HR^2^ (95% CI)	Cases	Adjusted HR^2^ (95% CI)
Height (cm)								
<159	18	Reference	20	Reference	19	Reference	74	Reference
159–165	13	0.58 (0.27–1.25)	38	1.44 (0.82–2.55)	29	1.26 (0.69–2.32)	58	0.89 (0.61–1.28)
>165	16	1.01 (0.48–2.13)	31	1.49 (0.80–2.76)	23	1.29 (0.66–2.53)	41	0.97 (0.63–1.51)
Missing	2	–	5	–	9	–	16	–
*p*_trend_		0.27		0.39		0.71		0.79
Weight (kg)								
<60	9	Reference	38	Reference	12	Reference	48	Reference
60–69	15	1.29 (0.56–3.00)	29	0.63 (0.38–1.03)	22	1.63 (0.79–3.34)	49	0.76 (0.51–1.14)
>69	23	1.88 (0.85–4.16)	22	0.52 (0.30–0.90)	37	2.77 (1.40–5.48)	77	1.12 (0.77–1.64)
Missing	2	–	5	–	9	–	15	–
*p*_trend_		0.25		0.04		0.001		0.10
BMI (kg/m^2^)^4^								
Underweight^5^	1	–	6	–	–	–	2	–
Normal weight	13	Reference	45	Reference	29	Reference	68	Reference
Overweight	22	2.15 (1.06–4.38)	33	1.08 (0.68–1.72)	30	1.44 (0.85–2.43)	61	0.96 (0.67–1.38)
Obese	11	2.66 (1.15–6.19)	5	0.50 (0.20–1.28)	12	1.41 (0.70–2.83)	42	1.31 (0.86–2.00)
Missing	2	–	5	–	9	–	16	–
*p*_trend_		0.09		0.003		0.56		0.49
Hip circumference (cm)								
<96	14	Reference	37	Reference	13	Reference	43	Reference
96–104	14	0.78 (0.36–1.70)	32	0.80 (0.49–1.31)	29	1.83 (0.94–3.56)	53	0.82 (0.54–1.24)
>104	18	1.11 (0.53–2.34)	17	0.51 (0.28–0.95)	26	1.97 (0.98–3.95)	63	0.94 (0.62–1.44)
Missing	3	–	8	–	12	–	30	–
*p*_trend_		0.64		0.10		0.13		0.59
Waist circumference (cm)								
<74	7	Reference	29	Reference	10	Reference	35	Reference
74–84	16	1.62 (0.65–4.08)	35	0.71 (0.42–1.19)	25	1.59 (0.75–3.36)	50	0.79 (0.51–1.24)
>84	23	2.81 (1.13–6.96)	22	0.55 (0.30–0.99)	34	2.55 (1.22–5.33)	74	0.97 (0.62–1.53)
Missing	3	–	8	–	11	–	30	–
*p*_trend_		0.06		0.13		0.03		0.47
Waist‐to‐hip ratio								
<0.76	4	Reference	23	Reference	10	Reference	23	Reference
0.76–0.82	22	3.83 (1.28–11.44)	37	0.90 (0.52–1.56)	25	1.50 (0.71–3.17)	69	1.54 (0.94–2.51)
>0.82	20	5.39 (1.74–16.72)	26	0.73 (0.39–1.34)	33	2.50 (1.19–5.25)	67	1.49 (0.89–2.48)
Missing	3	–	8	–	12	–	30	–
*p*_trend_		0.01		0.57		0.03		0.21
Waist‐to‐height ratio								
<0.45	5	Reference	23	Reference	9	Reference	23	Reference
0.45–0.52	18	1.87 (0.67–5.19)	41	0.76 (0.44–1.31)	31	1.53 (0.72–3.26)	55	0.91 (0.54–1.52)
>0.52	23	3.50 (1.24–9.93)	20	0.53 (0.27–1.03)	29	2.05 (0.93–4.51)	80	1.27 (0.75–2.16)
Missing	3	–	10	–	11	–	31	–
*p*_trend_		0.03		0.17		0.19		0.20

1 Sex‐specific tertiles were used in the analyses except for BMI.

2 Stratified on age, center and adjusted for smoking, and education level.

3 Stratified on age, center and adjusted for smoking, education level, and alcohol intake.

4 Underweight (BMI < 18.5), normal weight (18.5 ≤ BMI < 25), overweight (25 ≤ BMI < 30) and obese (BMI ≥30).

5 We excluded underweight group from the analysis due to few number of cases.

We observed inverse associations between some anthropometric variables and ESCC, including weight and HC in both men and women (Tables 1 and 2) and specifically BMI in men (HR 0.52, 95% CI: 0.28–0.95 for obese *vs*. normal weight; *p*‐value for interaction by sex = 0.009; Table 1), and WC in women (HR 0.55, 95% CI: 0.30–0.99 for WC >84 *vs*. <74 cm), although the *p‐*value for interaction by sex was not statistically significant (Table 2).

WHR was positively associated with GC in both men and women, although the association was not statistically significant in men (HR 1.57, 95% CI: 0.97–2.53 for WHR >0.96 *vs*. <0.91 and HR 2.50, 95% CI: 1.19–5.25 for WHR >0.82 *vs*. <0.76, respectively; Tables 1 and 2). In addition, weight and WC were positively associated with GC in women (Table 2). For GNC, there was an inverse association between height and GNC in men (HR 0.49, 95% CI: 0.30–0.81 for height >178 *vs*. <171 cm) but not in women (HR 0.97, 95% CI: 0.63–1.51 for height >165 *vs*. <159 cm; Tables 1 and 2).

BMI and WHR were moderately correlated (*r* = 0.43), as were WC and HC (*r* = 0.67). Upon adjustment for WHR, BMI was no longer significantly associated with EA in men or women (HR 1.21, 95% CI: 0.75–1.97 and HR 1.93, 95% CI: 0.80–4.68 for obese *vs*. normal weight, respectively; Tables 3 and 4). Conversely, the positive association observed for WC and WHR in relation to EA remained significant in both men and women after adjustment for HC and BMI, respectively (Tables 3 and 4). Furthermore, the positive association observed between HC and EA in men was attenuated after adjustment for WC (Table 3). For ESCC, the inverse association observed with BMI and HC in men remained significant after adjustment for WHR and WC, respectively (Table 3). In contrast, a positive association was observed in men for ESCC with WC adjusted for HC (HR 2.14, 95% CI: 1.06–4.32 for WC >98 *vs*. <90 cm) and WHR adjusted for BMI (HR 2.24, 95% CI: 1.16–4.32 for WHR >0.96 *vs*. <0.91; Table 3). While in women, the inverse association observed for ESCC with WC and HC was no longer significant after mutual adjustment (Table 4).

**Table 3:** Adjusted hazard ratios for esophageal and gastric cancer by subtype and subsite in men (*n* = 142,241) according to anthropometric factors (mutually adjusted) in the EPIC study

	Esophageal cancer				Gastric cancer			
	Adenocarcinoma		Squamous cell carcinoma		Cardia		Noncardia	
Men^1^	Cases	Adjusted HR^2^ (95% CI)	Cases	Adjusted HR^3^ (95% CI)	Cases	Adjusted HR^2^ (95% CI)	Cases	Adjusted HR^2^ (95% CI)
BMI (kg/m^2^)^4^ adjusted for waist‐to‐hip ratio								
Underweight^5^	–	–	1	–	1	–	1	–
Normal weight	50	Reference	53	Reference	51	Reference	49	Reference
Overweight	83	0.88 (0.60–1.28)	31	0.31 (0.19–0.50)	90	1.06 (0.73–1.55)	99	1.15 (0.79–1.67)
Obese	37	1.21 (0.75–1.97)	16	0.37 (0.19–0.71)	20	0.75 (0.42–1.34)	32	1.15 (0.69–1.92)
Missing	1		–		1		3	
*p*_trend_		0.47		<0.0001		0.51		0.83
Waist circumference (cm) adjusted for hip circumference								
<90	30	Reference	29	Reference	32	Reference	39	Reference
90–98	50	1.41 (0.85–2.32)	36	1.38 (0.80–2.36)	66	1.87 (1.16–3.00)	73	1.31 (0.84–2.05)
>98	75	2.67 (1.52–4.72)	31	2.14 (1.06–4.32)	55	1.99 (1.10–3.59)	55	0.90 (0.51–1.59)
Missing	16	–	5	–	10	–	17	–
*p*_trend_		0.001		0.11		0.03		0.14
Hip circumference (cm) adjusted for waist circumference								
<98	35	Reference	48	Reference	54	Reference	49	Reference
98–103	67	1.28 (0.80–2.04)	30	0.42 (0.25–0.73)	53	0.68 (0.44–1.05)	65	1.06 (0.69–1.63)
>103	52	0.82 (0.47–1.45)	17	0.17 (0.08–0.37)	45	0.62 (0.36–1.06)	51	0.92 (0.53–1.58)
Missing	17	–	6	–	11	–	19	–
*p*_trend_		0.08		<0.0001		0.15		0.79
Waist‐to‐hip ratio adjusted for BMI								
<0.91	21	Reference	18	Reference	25	Reference	32	Reference
0.91–0.96	52	1.82 (1.06–3.11)	36	1.57 (0.87–2.84)	63	1.37 (0.84–2.23)	81	1.33 (0.86–2.06)
>0.96	81	3.47 (1.99–6.06)	41	2.24 (1.16–4.32)	64	1.60 (0.94–2.71)	52	0.81 (0.49–1.35)
Missing	17	–	6	–	11	–	19	–
*p*_trend_		<0.0001		0.05		0.16		0.02

1 Sex‐specific tertiles were used in the analyses except for BMI.

2 Stratified on age, center and adjusted for smoking and education level.

3 Stratified on age, center and adjusted for smoking, education level and alcohol intake.

4 Underweight (BMI < 18.5), normal weight (18.5 ≤ BMI < 25), overweight (25 ≤ BMI < 30) and obese (BMI ≥30).

5 We excluded underweight group from the analysis due to few number of cases.

**Table 4:** Adjusted hazard ratios for esophageal and gastric cancer by subtype and subsite in women (*n* = 333,919) according to anthropometric factors (mutually adjusted) in the EPIC study

	Esophageal cancer				Gastric cancer			
	Adenocarcinoma		Squamous cell carcinoma		Cardia		Noncardia	
Women^1^	Cases	Adjusted HR^2^ (95% CI)	Cases	Adjusted HR^3^ (95% CI)	Cases	Adjusted HR^2^ (95% CI)	Cases	Adjusted HR^2^ (95% CI)
BMI (kg/m^2^)^4^ adjusted for waist‐to‐hip ratio								
Underweight^5^	1	–	6	–	–	–	2	–
Normal weight	13	Reference	45	Reference	29	Reference	68	Reference
Overweight	22	1.76 (0.85–3.64)	33	1.09 (0.67–1.76)	30	1.20 (0.70–2.07)	61	0.92 (0.63–1.33)
Obese	11	1.93 (0.80–4.68)	5	0.51 (0.20–1.34)	12	1.05 (0.50–2.19)	42	1.23 (0.79–1.93)
Missing	2	–	5	–	9	–	16	–
*p*_trend_		0.29		0.004		0.92		0.53
Waist circumference (cm) adjusted for hip circumference								
<74	7	Reference	29	Reference	10	Reference	35	Reference
74–84	16	2.44 (0.91–6.58)	35	0.78 (0.44–1.38)	25	1.39 (0.62–3.14)	50	0.86 (0.52–1.41)
>84	23	5.67 (1.76–18.26)	22	0.74 (0.34–1.59)	34	2.29 (0.92–5.72)	74	1.06 (0.59–1.93)
Missing	3	–	8	–	11	–	30	–
*p*_trend_		0.01		0.65		0.15		0.57
Hip circumference (cm) adjusted for waist circumference								
<96	14	Reference	37	Reference	13	Reference	43	Reference
96–104	14	0.44 (0.19–1.05)	32	0.89 (0.52–1.55)	29	1.43 (0.68–3.02)	53	0.83 (0.51–1.33)
>104	18	0.39 (0.15–1.05)	17	0.61 (0.28–1.34)	26	1.20 (0.50–2.90)	63	0.88 (0.50–1.55)
Missing	3	–	8	–	12	–	30	–
*p*_trend_		0.12		0.45		0.58		0.73
Waist‐to‐hip ratio adjusted for BMI								
<0.76	4	Reference	23	Reference	10	Reference	23	Reference
0.76–0.82	22	3.58 (1.19–10.81)	37	0.98 (0.56–1.72)	25	1.43 (0.68–3.05)	69	1.56 (0.95–2.56)
>0.82	20	4.40 (1.35–14.33)	26	0.91 (0.47–1.74)	33	2.34 (1.07–5.12)	67	1.48 (0.86–2.55)
Missing	3	–	8	–	12	–	30	–
*p*_trend_		0.04		0.94		0.07		0.23

1 Sex‐specific tertiles were used in the analyses except for BMI.

2 Stratified on age, center and adjusted for smoking, and education level.

3 Stratified on age, center and adjusted for smoking, education level, and alcohol intake.

4 Underweight (BMI <18.5), normal weight (18.5 ≤ BMI < 25), overweight (25 ≤ BMI < 30) and obese (BMI ≥30).

5 We excluded underweight group from the analysis due to few number of cases.

After adjustment for BMI, WHR remained positively associated with GC in women (Table 4). A positive association was found between WC and GC in men after adjustment for HC (HR 1.99, 95% CI: 1.10–3.59 for >98 *vs*. <90 cm; Table 3). While the positive association observed for GC with WC in women was attenuated after adjustment for HC (HR 2.29, 95% CI: 0.92–5.72 for WC >84 *vs*. <74 cm; Table 4). For GNC, results did not change when we mutually adjusted for BMI and WHR or when WC and HC were mutually adjusted in either men or women (Tables 3 and 4).

No statistically significant interactions were observed between BMI or smoking status and any of the outcomes.

### Reproductive factors

Baseline characteristics of women according to OC and menopausal hormonal use are presented in Supporting Information Table S2. Women who reported using OC pills were younger, had a slightly lower WC and HC, were more educated, more likely to be smokers and more physically active, had a lower intake of fruits and vegetables and lower prevalence of diabetes compared to nonusers of OC. While women who reported using hormones for menopause were slightly older and less educated than nonusers.

We found no associations between reproductive factors and ESCC or GC (Table 5). However, an inverse association was observed between parity and EA (HR 0.38, 95% CI: 0.14–0.99 for >2 *vs*. 0 pregnancies). For GNC, compared to women who had their first pregnancy at an earlier age (<22 years), women who had their pregnancy at a later age (>26 years) had a lower risk of GNC (HR 0.54, 95% CI: 0.32–0.91). In addition, compared to women who had not undergone ovariectomy, women who had a bilateral ovariectomy had a higher risk of GNC (HR 1.87, 95% CI: 1.04–3.36), although there were only 13 cases in this group.

**Table 5:** Adjusted hazard ratios for esophageal and gastric cancer by subtype and subsite according to reproductive factors in women (*n* = 333,919) in the EPIC study

	Esophageal cancer				Gastric cancer			
	Adenocarcinoma		Squamous cell carcinoma		Cardia		Noncardia	
	Cases	Adjusted HR^1^ (95% CI)	Cases	Adjusted HR^2^ (95% CI)	Cases	Adjusted HR^1^ (95% CI)	Cases	Adjusted HR^1^ (95% CI)
Age at menarche, years								
<12	3	0.38 (0.11–1.24)	13	1.12 (0.60–2.07)	8	0.77 (0.36–1.63)	27	1.18 (0.77–1.83)
12–14	38	Reference	60	Reference	52	Reference	117	Reference
>14	6	0.50 (0.21–1.21)	17	0.83 (0.48–1.44)	16	0.93 (0.53–1.64)	33	0.91 (0.61–1.35)
Missing	2	–	4	–	4	–	12	–
*p*_trend_		0.10		0.71		0.78		0.63
Duration of menstrual cycle, years								
<30	5	Reference	14	Reference	15	Reference	36	Reference
30–35	15	1.74 (0.57–5.35)	29	1.02 (0.52–2.01)	16	0.56 (0.26–1.22)	63	1.23 (0.76–1.99)
>35	16	1.46 (0.48–4.48)	34	1.14 (0.58–2.24)	37	1.11 (0.57–2.18)	63	1.04 (0.64–1.69)
Missing	13	–	17	–	12	–	27	–
*p*_trend_		0.62		0.90		0.01		0.56
Ever been pregnant								
No	6	Reference	11	Reference	5	Reference	18	Reference
Yes	41	0.72 (0.30–1.72)	80	0.99 (0.51–1.91)	74	1.56 (0.63–3.89)	158	0.95 (0.58–1.56)
Missing	2	–	3	–	1	–	13	–
Age at first pregnancy, years								
<22	6	Reference	22	Reference	22	Reference	38	Reference
22–26	13	1.18 (0.43–3.23)	25	0.54 (0.29–1.01)	25	0.70 (0.38–1.27)	55	0.65 (0.42–1.00)
>26	14	1.99 (0.70–5.59)	15	0.53 (0.26–1.10)	15	0.81 (0.40–1.64)	31	0.54 (0.32–0.91)
Missing	16	–	32	–	18	–	65	–
*p*_trend_		0.30		0.11		0.50		0.05
Parity (number of full‐term pregnancies)								
0	8	Reference	13	Reference	7	Reference	19	Reference
1–2	27	0.69 (0.31–1.54)	57	1.16 (0.62–2.18)	47	1.34 (0.60–2.99)	94	1.03 (0.62–1.70)
>2	10	0.38 (0.14–0.99)	18	0.66 (0.32–1.38)	23	1.10 (0.47–2.59)	58	1.05 (0.62–1.80)
Missing	4	–	6	–	3	–	18	–
*p*_trend_		0.12		0.13		0.64		0.98
Number of live‐born children								
1	9	Reference	17	Reference	10	Reference	15	Reference
2–3	23	0.54 (0.25–1.18)	44	0.74 (0.41–1.35)	38	0.79 (0.39–1.61)	64	0.88 (0.50–1.57)
>3	4	0.34 (0.09–1.31)	6	0.52 (0.19–1.40)	10	1.15 (0.46–2.88)	16	0.91 (0.43–1.93)
Missing	13	–	27	–	22	–	94	–
*p*_trend_		0.18		0.40		0.54		0.91
Ever breastfeed								
No	14	Reference	20	Reference	19	Reference	43	Reference
Yes	29	0.63 (0.33–1.22)	61	1.07 (0.64–1.81)	54	0.86 (0.51–1.47)	120	0.83 (0.58–1.19)
Missing	6	–	13	–	7	–	26	–
Duration of breastfeeding (months)								
<4	11	Reference	20	Reference	20	Reference	31	Reference
4–10	8	0.72 (0.28–1.86)	20	1.01 (0.52–1.95)	18	0.86 (0.44–1.67)	42	1.17 (0.72–1.89)
>10	10	0.78 (0.31–1.94)	20	1.24 (0.63–2.43)	16	0.90 (0.45–1.83)	47	1.25 (0.76–2.06)
Missing	20	–	34	–	26	–	69	–
*p*_trend_		0.77		0.77		0.90		0.67
Menopause status								
Pre/perimenopausal	9	Reference	18	Reference	23	Reference	49	Reference
Postmenopausal	36	2.12 (0.76–5.89)	73	2.14 (0.95–4.79)	51	0.83 (0.39–1.76)	127	1.31 (0.72–2.38)
Surgical menopause	4	3.66 (0.96–13.96)	3	1.45 (0.35–5.95)	6	1.70 (0.60–4.85)	13	2.26 (0.99–4.95)
Age at menopause, years								
<48	10	Reference	19	Reference	13	Reference	35	Reference
48–51	14	1.34 (0.58–3.13)	26	1.37 (0.74–2.55)	19	1.27 (0.62–2.60)	55	1.34 (0.87–2.07)
>51	4	0.51 (0.16–1.70)	18	1.20 (0.60–2.38)	15	1.34 (0.62–2.93)	34	0.98 (0.60–1.59)
Missing	21	–	31	–	33	–	65	–
*p*_trend_		0.24		0.61		0.73		0.26
Ever use of hormones for menopause								
No	30	Reference	58	Reference	49	Reference	119	Reference
Yes	14	0.96 (0.49–1.89)	28	0.93 (0.57–1.53)	27	1.01 (0.61–1.67)	47	1.03 (0.71–1.50)
Missing	5	–	8	–	4	–	23	–
Duration of hormone use for menopause (years)								
<2	7	Reference	12	Reference	11	Reference	15	Reference
≥2	7	0.85 (0.28–2.62)	10	0.76 (0.30–1.94)	13	0.88 (0.37–2.06)	24	1.40 (0.69–2.84)
Missing	35	–	72	–	56	–	150	–
Ever use of OC pill use								
No	25	Reference	45	Reference	36	Reference	102	Reference
Yes	21	0.81 (0.41–1.58)	44	1.02 (0.61–1.69)	41	1.07 (0.64–1.78)	74	0.93 (0.66–1.33)
Missing	3	–	5	–	3	–	13	–
Duration of OC pill use (years)								
<5	8	Reference	16	Reference	13	Reference	28	Reference
≥5	12	0.92 (0.37–2.32)	22	1.05 (0.52–2.11)	26	1.63 (0.81–3.30)	39	1.03 (0.61–1.73)
Missing	29	–	56	–	41	–	122	–
Ovariectomy								
No	35	Reference	66	Reference	58	Reference	119	Reference
Unilateral	–	–	3	0.51 (0.13–2.11)	3	0.80 (0.25–2.55)	12	1.64 (0.88–3.06)
Bilateral	4	1.94 (0.68–5.54)	3	0.73 (0.22–2.42)	6	1.98 (0.84–4.65)	13	1.87 (1.04–3.36)
Missing	10	–	22	–	13	–	45	–

1 Stratified on age, center and adjusted for smoking, BMI and education level.

2 Stratified on age, center and adjusted for smoking, BMI, education level and alcohol intake.

Exclusion of esophageal and gastric cancer cases diagnosed in the first 2 years of follow‐up did not substantially change the associations observed for anthropometric or reproductive factors (data not shown). In addition, analyses restricted to measured anthropometric factors yielded similar results to those when participants with self‐reported data were included (data not shown). Finally, comparing the baseline characteristics of included participants to those excluded from the analysis because they lacked dietary or lifestyle information, revealed no substantial differences between the two groups, except participants excluded were very slightly older than the included participants.

## Discussion

In this large prospective study, abdominal obesity was positively associated with EA and GC, while the findings for ESCC were less clear. No associations were observed for GNC either with general or abdominal obesity. With regards to reproductive factors in women, there were inverse associations between parity and EA and between age at first pregnancy and GNC; whereas a positive association was observed for bilateral ovariectomy and GNC.

A number of meta‐analyses have shown that BMI was positively associated with EA and GC.^11^, ^12^, ^32^ Our study found a positive association between BMI and EA but these associations attenuated after adjustment for WHR, and no significant association was observed between BMI and GC independently of WHR. The NIH‐AARP Diet and Health Study, a prospective cohort study of equivalent size to EPIC, showed a nonsignificant positive association between BMI and EA (HR 1.77, 95% CI: 0.90–3.49; for BMI ≥35 *vs*. 18.5 to <25) and a significant positive association between BMI and GC (HR 3.28, 95% CI: 1.76–6.11 for BMI ≥35 *vs*. 18.5 to <25) after adjustment for WHR.^15^ A nested case–control study also reported no association between BMI and EA after adjustment for abdominal diameter.^33^

In our study, WHR and WC were positively associated with EA independently of BMI and HC. WHR was also positively associated with GC in women only independently of BMI, and WC was positively associated with GC in men after adjustment for HC. The NIH‐AARP Diet and Health Study showed a positive association between WHR and EA (HR 1.17, 95% CI: 0.99–2.18; for Quartile 4 *vs*. Quartile 1), but no association between WHR and GC (HR 1.08, 95% CI: 0.71–1.63; for Quartile 4 *vs*. Quartile 1) when adjusted for BMI.^15^ This cohort study also showed a positive association between WC and risk of EA and GC, after adjustment for HC.^15^ In addition, two other cohort studies showed a positive association between WC and EA but these findings were not adjusted for HC.^33^, ^34^ A recent meta‐analysis of six prospective studies reported positive associations for GC with WC but not with WHR.^35^

Taken together, our findings show that WC and WHR rather than BMI appears to be more closely associated with EA and GC. These associations could potentially be explained by mechanical effects of obesity, especially abdominal obesity‐promoting GERD, which is associated with an increased risk of EA and GC.^16^, ^33^

In our study, the findings for ESCC were less clear; for example, in men, HC and BMI were inversely associated with ESCC, while WC and WHR were positively associated. A number of prospective cohort studies have reported an inverse association between BMI and ESCC.^2^, ^7^, ^14^ Few epidemiological studies have examined the association between abdominal obesity and ESCC and those that have reported no associations.^33^, ^34^ The underlying mechanisms for the observed associations between adiposity and ESCC are not well‐known and need to be further investigated but our study sheds further light on the contrasting observations for anthropometric measures and ESCC.

Our study found no association for GNC with BMI, WC or WHR, which is in line with previous meta‐analyses.^32^, ^35^ However, we observed an inverse association between height and GNC in men only. Previous cohort studies that examined the association between height and GNC showed a nonsignificant inverse association.^7^, ^15^, ^36^ The observed association could potentially be explained by the positive association between GNC and *Helicobacter pylori* infection, which may cause poor growth during childhood and has been associated with lower socioeconomic status,^37^, ^38^ which is in turn related to poor nutrition and leads to shorter adult height.^39^

With respect to reproductive factors, we found no associations between reproductive factors and ESCC or GC. However, an inverse association was observed between parity and EA. A meta‐analysis by Wang *et al*.^21^ reported no association for EA with reproductive factors; however, this analysis did show an inverse association between breastfeeding and EA.^21^ A recent meta‐analysis reported an inverse association between breastfeeding, parity and esophageal cancer.^22^

Few epidemiological studies have studied the association between age at first pregnancy and upper gastrointestinal cancers. The Women's Health Initiative Study reported no association between age at first pregnancy and ESCC but this was restricted to postmenopausal women.^26^ Furthermore, a population‐based case–control study showed no association between age at first pregnancy and distal gastric cancer (odds ratio: 0.83, 95% CI: 0.44–1.57, for ≥25 years *vs*. nulliparous).^40^ No other study has reported an inverse association between age at first pregnancy and GNC.

Our updated analysis with longer follow‐up confirms the previously reported positive association between bilateral ovariectomy and GNC.^28^ While the NIH‐AARP Diet and Health Study reported a nonsignificant increased risk for EA and GC combined (*n* = 65 cases) in women who had undergone ovariectomy,^27^ the Million Women's Study Cohort reported no association with GC or GNC.^25^ As estrogen has been suggested as a protective factor for EA and gastric cancer,^41^, ^42^ ovariectomy and consequently decreased estrogen levels could explain the increased risk of GNC in these individuals. Interestingly, studies in animal models have shown that ovariectomized female mice had an increased risk of gastric cancer.^41^

Our results suggest hormonal factors may play a role in the etiology of upper gastrointestinal cancers but the underlying biology is not defined. For gastric cancer, estrogens may protect against the development of this malignancy by acting on estrogen receptors (ERα and ERβ), which have been identified in gastric cancer cells.^43^ Estrogen inhibits cell growth and increases apoptosis in gastric cancer cells^44^, ^45^ and stimulates the expression of trefoil factor proteins, which play a role in mucosal protection and repair and their trefoil factor genes may act as tumor suppressors.^41^

Estrogen regulates body adiposity and fat distribution through ERs in the brain, and by interacting with leptin pathways.^46^ Body fat distribution varies by sex as men tend to accrue more visceral fat, while women accrue more fat in the subcutaneous depot.^47^ Estrogen promotes the accumulation of subcutaneous fat^48^ and the decrease in estrogen levels in menopausal women is associated with an increase in visceral fat.^49^ The accumulation of visceral fat is associated with an increased risk of esophageal and gastric cancer.^35^ Hence, estrogen regulation of leptin levels in women may play a protective role directing accumulation of subcutaneous fat over visceral fat and consequently may explain the sex differences in the incidence of esophageal and gastric cancer.

However, any potential protective mechanisms associated with estrogen are in conflict with the fact that obesity, which has been linked with an increased risk of some upper gastrointestinal cancers, is associated with higher estrogen levels. Nevertheless, the associations observed in our study were site‐specific, in that abdominal obesity was positively associated with EA and GC (and not GNC) and bilateral ovariectomy were only positively associated with GNC. Furthermore, in addition to the effect on sex hormones, there are other biological effects of obesity, including insulin resistance, where levels of insulin and bioavailability of IGF are increased, which promotes cell division and inhibits apoptosis.^17^ Obesity also increases concentrations of adipokines (e.g., leptin) and proinflammatory cytokines (e.g., tumor necrosis factor‐α), which may contribute to cancer development.^17^, ^18^

Strengths of our study include its prospective study design, large sample size and availability of standardized information on reproductive factors, and potential confounders. In addition, anthropometric factors were mostly measured by trained professionals. Due to the prospective nature of the study, the likelihood of recall and selection bias is minimal. The large sample size allowed the analyses of esophageal and gastric cancer by subtype and subsite, and allowed analyses by gender; however, despite the size of the cohort and the long‐term follow‐up, the number of cases in some analyses was quite small. Our study has some limitations. We used a single assessment of anthropometric and reproductive factors collected at baseline as data during follow‐up was not available; if anthropometric and reproductive factors changed during follow‐up, this could lead to some misclassification and possibly bias the results toward null. Although we adjusted for several potential confounders, we lacked information on GERD and *Helicobacter pylori* infection, which have been associated with EA and GC, respectively; therefore, residual confounding cannot be excluded. We also lacked data on the reason for ovariectomy in the women who underwent this procedure. In addition, EPIC cohort participants are of European descent, which limits the generalizability of our findings to other ethnicities. Finally, as we analyzed anthropometric and reproductive factors in relation to both esophageal and gastric cancer by subtype and subsite, some of our associations may have arisen by chance as a result of multiple comparisons.

In conclusion, the results of our study suggest that abdominal obesity may influence risk for EA and GC. Furthermore, some reproductive factors in women may influence risk for EA and GNC, specifically. Together, these findings may support a role for hormonal pathways in upper gastrointestinal cancer development; however, in order to fully investigate these pathways, future studies should investigate endogenous hormone measurements in relation to these cancers. Considering these results, maintaining a healthy weight should be suggested as an evidence‐based lifestyle recommendation for EA and GC prevention.

## Author contributions

Study concept, study design and data management: AJC. Statistical analysis: HS. Article drafting—first version, interpretation of results and critical article review: AJC and HS. Data contribution and final version of the article: all EPIC co‐authors.

## Data sharing

For information on how to submit an application for gaining access to EPIC data and/or biospecimens, please follow the instructions at http://epic.iarc.fr/access/index.php.

## Supporting information


**Table S1** Baseline characteristics of men and women according to BMI categories in the EPIC study
**Table S2**. Baseline characteristics of women by OC and menopausal hormonal use in the EPIC study
