# Supplementary material for: Anthropometric and reproductive factors and risk of esophageal and gastric cancer by subtype and subsite: Results from the European Prospective Investigation into Cancer and Nutrition (EPIC) cohort
Source: Int J Cancer. 2019 May 21;146(4):929–42. doi: 10.1002/ijc.32386 (PMC6973006; doi:10.1002/ijc.32386)
Supplement: Supplementary file 1 — Table S1 Baseline characteristics of men and women according to BMI categories in the EPIC study Table S2. Baseline characteristics of women by OC and menopausal hormonal use in the EPIC study [file IJC-146-929-s001.docx]

Supplementary Table 1. Baseline characteristics of men and women according to BMI categories in the EPIC study

|  | |  | | |  | | |  | BMI (kg/m^2^)^a^ | | | | | | | | |  | | |
| --- | --- | --- | --- | --- | --- | --- | --- | --- | --- | --- | --- | --- | --- | --- | --- | --- | --- | --- | --- | --- |
|  |  | | | |  | | | Men | |  |  | | |  |  | Women |  | | |  |
|  |  | | | Underweight  (BMI <18.5) | | | | Normal weight  (18.5≤BMI<25) | | Overweight (25≤BMI<30) | | Obese  (BMI ≥30) | |  | Underweight  (BMI <18.5) | Normal weight  (18.5≤BMI<25) | Overweight (25≤BMI<30) | | | Obese  (BMI ≥30) |
|  |  | | | (n=567) | | | | (n=50,112) | | (n=68,737) | | (n=21,698) | |  | (n=4,432) | (n=129,729) | (n=78,695) | | | (n=37,504) |
| Age at recruitment^b^ (years) | | 49.6±15.4 | | | | | 50.5±11.2 | | | 53.0±9.4 | | 53.8±8.9 | |  | 45.5±13.1 | 48.9±10.9 | 53.0±9.8 | | 54.0±9.4 | |
| Waist circumference^b^ (cm) | |  | 74.4±5.8 | | | | 86.0±6.1 | | | 96.2±6.2 | 108.9±8.2 | | |  | 64.6±4.4 | 73.2±6.2 | 84.1±7.0 | | 97.7±9.7 | |
| Hip circumference^b^ (cm) | |  | 87.9±4.6 | | | | 95.7±4.6 | | | 101.6±4.7 | 109.7±6.6 | | |  | 87.2±4.4 | 95.6±5.2 | 103.9±5.3 | | 115.3±8.5 | |
| Education level (%) | |  |  | | | |  | | |  |  | | |  |  |  |  | |  | |
| None | |  | 2.9 | | | | 1.6 | | | 4.2 | 9.5 | | |  | 0.4 | 1.4 | 7.5 | | 18.0 | |
| Primary school | |  | 22.0 | | | | 22.8 | | | 32.2 | 39.7 | | |  | 12.2 | 19.5 | 34.2 | | 39.6 | |
| Technical/professional | |  | | 22.5 | | | 24.3 | | | 25.0 | 22.4 | | |  | 21.1 | 25.1 | 24.1 | | 19.4 | |
| Secondary school | |  | | 19.1 | | | 16.0 | | | 12.1 | 9.7 | | |  | 24.9 | 21.6 | 15.2 | | 10.6 | |
| University degree | |  | | 30.2 | | | 33.2 | | | 24.8 | 17.3 | | |  | 38.0 | 29.0 | 15.9 | | 9.7 | |
| Missing | |  | | 3.2 | | | 2.1 | | | 1.7 | 1.4 | | |  | 3.4 | 3.3 | 3.2 | | 2.7 | |
| Smoking (%) | |  | | |  | |  | | |  |  | | |  |  |  |  | |  | |
| Never | |  | | 33.3 | | | 35.3 | | | 29.3 | 26.1 | | |  | 48.7 | 48.0 | 53.9 | | 62.5 | |
| Former, quit ≤10 years | |  | | 5.6 | | | 9.4 | | | 13.7 | 17.2 | | |  | 6.2 | 8.6 | 8.3 | | 7.5 | |
| Former, quit 11-20 years | |  | | 6.5 | | | 8.9 | | | 12.3 | 13.0 | | |  | 5.2 | 7.4 | 6.7 | | 5.9 | |
| Former, quit 20+years | |  | | 7.8 | | | 10.1 | | | 11.8 | 10.5 | | |  | 5.5 | 6.5 | 6.7 | | 5.2 | |
| Current,1-15 cigarettes/day | |  | | 15.3 | | | 11.6 | | | 9.5 | 7.8 | | |  | 16.9 | 14.3 | 11.6 | | 8.6 | |
| Current,16-25 cigarettes/day | |  | | 14.5 | | | 8.8 | | | 7.9 | 7.9 | | |  | 7.0 | 5.6 | 5.6 | | 4.5 | |
| Current, 26+ cigarettes/day | |  | | 4.6 | | | 3.2 | | | 3.7 | 5.2 | | |  | 1.0 | 1.0 | 1.1 | | 1.2 | |
| Current or occasional pipe/cigar | |  | | 8.3 | | | 9.1 | | | 8.5 | 9.0 | | |  | 7.3 | 6.1 | 3.9 | | 2.7 | |
| Missing | |  | | 4.1 | | | 3.6 | | | 3.3 | 3.3 | | |  | 2.2 | 2.4 | 2.2 | | 2.0 | |
| Physical activity (%) | |  | |  | | |  | | |  |  | | |  |  |  |  | |  | |
| Inactive | | 29.8 | | | |  | 15.9 | | | 18.9 | 24.5 | | |  | 20.4 | 18.3 | 28.2 | | 40.4 | |
| Moderately inactive | |  | | 29.8 | | | 30.5 | | | 30.9 | 29.9 | | |  | 36.7 | 35.6 | 34.3 | | 31.0 | |
| Moderately active | |  | | 20.1 | | | 24.9 | | | 24.2 | 23.3 | | |  | 24.8 | 25.2 | 20.4 | | 16.2 | |
| Active | |  | | 16.8 | | | 26.0 | | | 24.0 | 20.9 | | |  | 16.6 | 19.3 | 15.6 | | 11.2 | |
| Missing | |  | | 3.5 | | | 2.7 | | | 2.0 | 1.4 | | |  | 1.5 | 1.6 | 1.5 | | 1.2 | |
| Energy intake^b^ (kcal/day) | |  | | 2359±679 | | | 2411±650 | | | 2401±655 | | | 2443±709 |  | 1948±531 | 1946±519 | 1913±530 | | 1906±562 | |
| Vegetable intake^b^ (g/day) | |  | | 175±138 | | | 178±134 | | | 196±153 | | | 222±175 |  | 222±155 | 212±139 | 217±147 | | 236±159 | |
| Fruit intake^b^ (g/day) | |  | | 165±182 | | | 188±166 | | | 212±188 | | | 232±202 |  | 229±199 | 242±177 | 261±187 | | 273±194 | |
| Red meat intake^b^ (g/day) | |  | | 40±40 | | | 49±41 | | | 57±42 | | | 63±44 |  | 29±30 | 35±30.5 | 41±30 | | 42±31 | |
| Processed meat intake^b^ (g/day) | |  | | 36±42 | | | 38±37 | | | 43±39 | | | 47±45 |  | 20±23 | 24±23.4 | 26±25 | | 27±29 | |
| Alcohol intake^b^ (g/day) | |  | | 15±22 | | | 18±21 | | | 21±23 | | | 23±27 |  | 7±11 | 9±12 | 8±11 | | 5±10 | |
| History of diabetes (%) | |  | | 1.4 | | | 2.4 | | | 3.9 | | | 7.6 |  | 1.0 | 1.1 | 2.9 | | 7.0 | |

^a^84,686 individuals were missing BMI

^b^ Values are means ± standard deviations (SD)

Supplementary Table 2. Baseline characteristics of women by OC and menopausal hormonal use in the EPIC study

|  |  | OC use | |  | Menopausal hormone use | |
| --- | --- | --- | --- | --- | --- | --- |
|  | Total women | Yes | No |  | Yes | No |
|  | (n=333,919) |  |  |  |  |  |
| **Age at recruitment^a^ (years)** | 50.8±9.8 | 48.1±8.9 | 55.1±9.4 |  | 55.3±5.8 | 49.3±10.6 |
| **BMI^a^ (kg/m^2^)** | 25.4±4.6 | 24.7±4.3 | 26.5±4.8 |  | 25.3±4.1 | 25.5±4.7 |
| **Waist circumference^a^ (cm)** | 80.2±11.5 | 78.2±10.7 | 82.9±11.9 |  | 80.1±10.5 | 80.3±11.8 |
| **Hip circumference^a^ (cm)** | 101.1±9.3 | 99.8±8.8 | 102.8±9.7 |  | 100.8±8.5 | 101.2±9.5 |
| **Education level (%)** |  |  |  |  |  |  |
| None | 4.6 | 2.0 | 8.6 |  | 1.9 | 5.8 |
| Primary school | 24.2 | 17.6 | 33.8 |  | 23.9 | 24.0 |
| Technical/professional | 21.8 | 25.3 | 17.1 |  | 25.8 | 19.5 |
| Secondary school | 23.9 | 25.4 | 21.2 |  | 24.7 | 24.7 |
| University degree | 23.0 | 27.5 | 16.4 |  | 20.4 | 23.7 |
| Missing | 2.5 | 2.2 | 2.9 |  | 3.3 | 2.3 |
| **Smoking (%)** |  |  |  |  |  |  |
| Never | 48.2 | 42.4 | 56.7 |  | 44.1 | 49.8 |
| Former, quit ≤ 10 years | 8.2 | 9.8 | 5.9 |  | 8.1 | 8.3 |
| Former, quit 11-20 years | 6.9 | 8.4 | 5.0 |  | 7.1 | 6.7 |
| Former, quit 20+ years | 6.6 | 7.0 | 6.2 |  | 8.9 | 5.7 |
| Current,1-15 cigarettes/day | 12.2 | 13.5 | 10.4 |  | 12.2 | 12.0 |
| Current,16-25 cigarettes/day | 5.4 | 6.3 | 4.1 |  | 5.9 | 5.2 |
| Current, 26+ cigarettes/day | 1.0 | 1.1 | 0.9 |  | 0.9 | 1.0 |
| Current or occasional pipe/cigar | 8.3 | 8.4 | 7.9 |  | 9.7 | 8.2 |
| Missing | 3.2 | 3.1 | 2.9 |  | 3.1 | 3.1 |
| **Physical activity (%)** |  |  |  |  |  |  |
| Inactive | 21.9 | 15.7 | 30.9 |  | 19.1 | 23.0 |
| Moderately inactive | 33.9 | 34.3 | 33.7 |  | 36.3 | 33.0 |
| Moderately active | 27.3 | 30.6 | 22.7 |  | 27.9 | 27.4 |
| Active | 15.2 | 17.6 | 11.6 |  | 15.5 | 14.9 |
| Missing | 1.7 | 1.8 | 1.1 |  | 1.2 | 1.7 |
| **Energy intake^a^ (kcal/day)** | 1932±540 | 1939±539 | 1933±541 |  | 1920±528 | 1943±545 |
| **Vegetable intake^a^ (g/day)** | 219±143 | 210±133 | 235±155 |  | 213±133 | 227±148 |
| **Fruit intake^a^ (g/day)** | 243±178 | 225±167 | 272±190 |  | 236±170 | 251±184 |
| **Red meat intake^a^ (g/day)** | 37±31 | 37±32 | 39±31 |  | 40±32 | 37±32 |
| **Processed meat intake^a^ (g/day)** 27±25 | | 28±26 | 24±24 |  | 28±24 | 25±25 |
| **Alcohol intake^a^ (g/day)** | 7±12 | 9±12 | 6±11 |  | 9±13 | 7±11 |
| **History of diabetes (%)** | 2.4 | 1.6 | 3.6 |  | 2.2 | 2.5 |

**^a^** Values are means ± standard deviations (SD)
